# Penetrating Head Injury With a Motor Bike Key

**DOI:** 10.7759/cureus.89714

**Published:** 2025-08-10

**Authors:** Piyush Kirange, Shantanu S Navgale, Sampada Wankhede, Satish B Dharap

**Affiliations:** 1 General Surgery, Topiwala National Medical College and Bai Yamunabai Laxman (BYL) Nair Charitable Hospital, Mumbai, IND

**Keywords:** bike key, ct brain, left lateral wall orbitotomy, multidisciplinary approach, penetrating head injury

## Abstract

Traumatic head injuries can be penetrating and non-penetrating. Penetrating head injuries are usually caused by low to high velocity, either with sharp or blunt objects. Penetrating objects may include knives, nails, forks, scissors, pencils, and even keys. Patients with penetrating brain injuries are mostly involved in road traffic accidents, assault injuries, accidental injuries, or even suicide attempts. This case report focuses on an accidental penetrating brain injury due to a bike key to a 21-year-old male near the left orbital wall. The patient underwent left lateral wall orbitotomy with removal of the foreign body within 12 hours of presentation to the emergency department. With proper multidisciplinary management, the postoperative course was uneventful with no neurological or ophthalmic complications. Prompt intervention in penetrating head injury is needed to get a good postoperative outcome.

## Introduction

Traumatic head injuries can be penetrating and non-penetrating. Penetrating head injuries are usually caused by low to high velocity, either with sharp or blunt objects. High velocity penetrating objects such as machine-gun bullets often cause fatal damage. Low velocity penetrating objects may include knives, nails, forks, scissors, pencil and even keys often as a result of homicidal or accidental or rarely suicidal impact. Low-velocity penetrating injuries, especially those involving atypical or household objects, are rarely encountered and are less frequently reported in the literature [1,2]. Despite its relatively blunt design, a bike key may cause significant damage under sufficient force, especially when directed toward vulnerable cranial areas.

We present a rare and unusual case of a penetrating head injury caused by a bike key, emphasizing the need for a thorough clinical and radiological assessment in all cases of cranial trauma, regardless of the perceived severity or mechanism of injury [3,4].

## Case presentation

**Figure 1:**
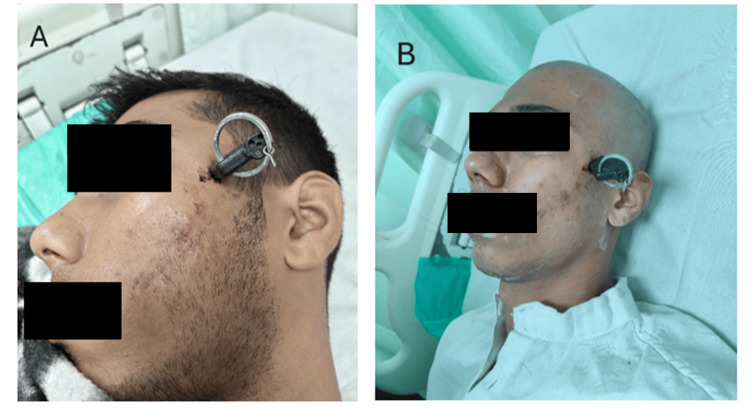
Clinical photographs of patient with penetrating bike key.

A 21-year-old male presented to the surgical emergency with a motorbike key impacted into the left side of his face, 5 cm above and anterior to the tragus. The incident occurred while he was getting down the stairs at his residence with the bike key in his hand, and he accidentally slipped from a height of 10 feet, resulting in a penetrating injury to the face (Figure 1).

At the time of presentation to the emergency room, he was haemodynamically stable with a Glasgow Coma Score of 15/15 with no focal neurological deficit. Ophthalmologic examination, which was done to rule out internal eyeball injury, suggested no abnormality. Blood investigations were within normal limits.

Radiological investigations, including the skull X-ray with both AP and lateral views as well as computed tomography (CT) brain plain with 3D CT face, were done to visualize the exact location of the penetrated portion of the key near the brain and left orbital cavity.

**Figure 2:**
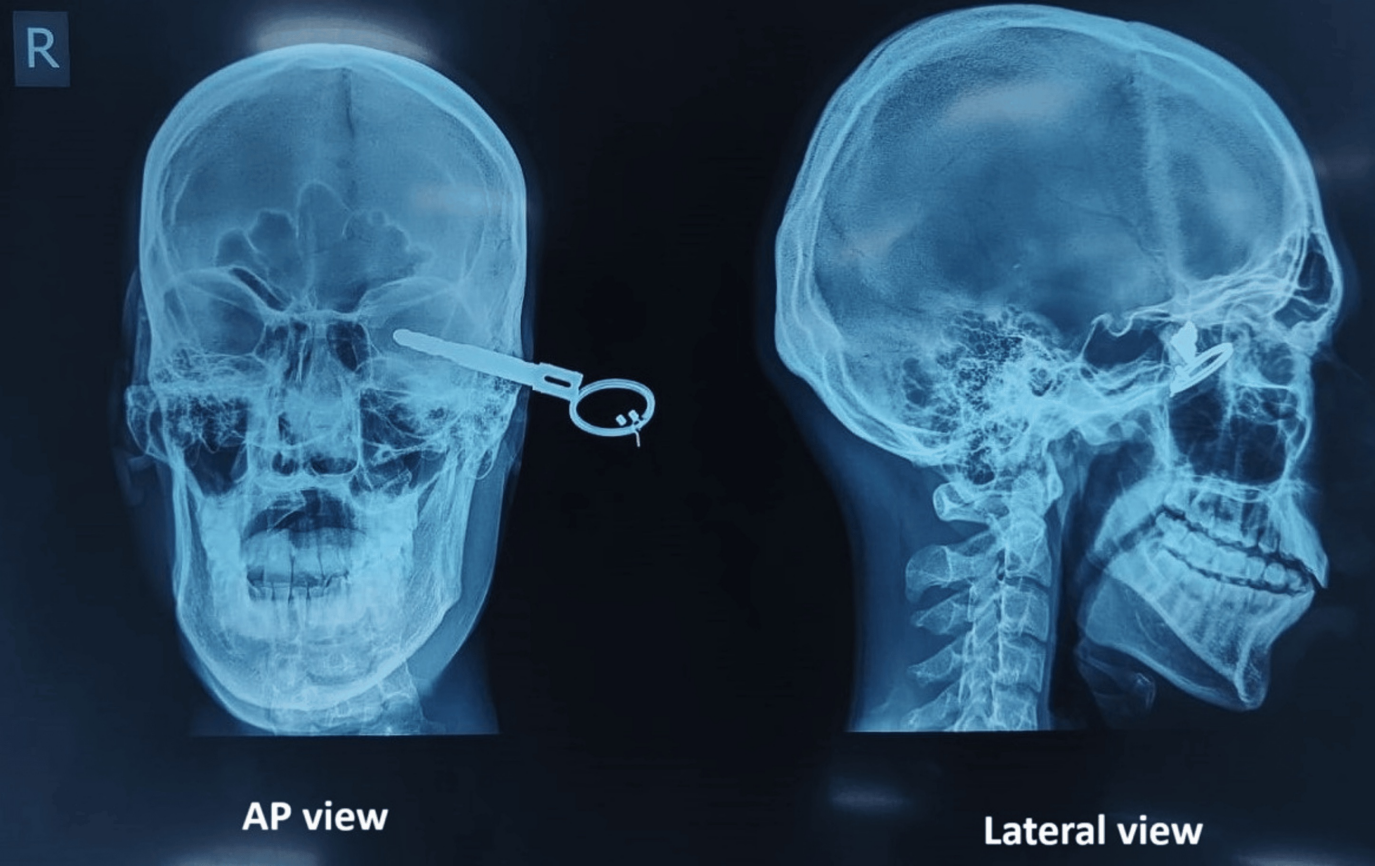
Plain X-ray AP view and lateral view showing the bike key in left orbit.

X-ray skull AP view showed a 7 mm deep intra-orbital cavity penetration of the bike key from the left lateral side of the face (Figure 2).

**Figure 3:**
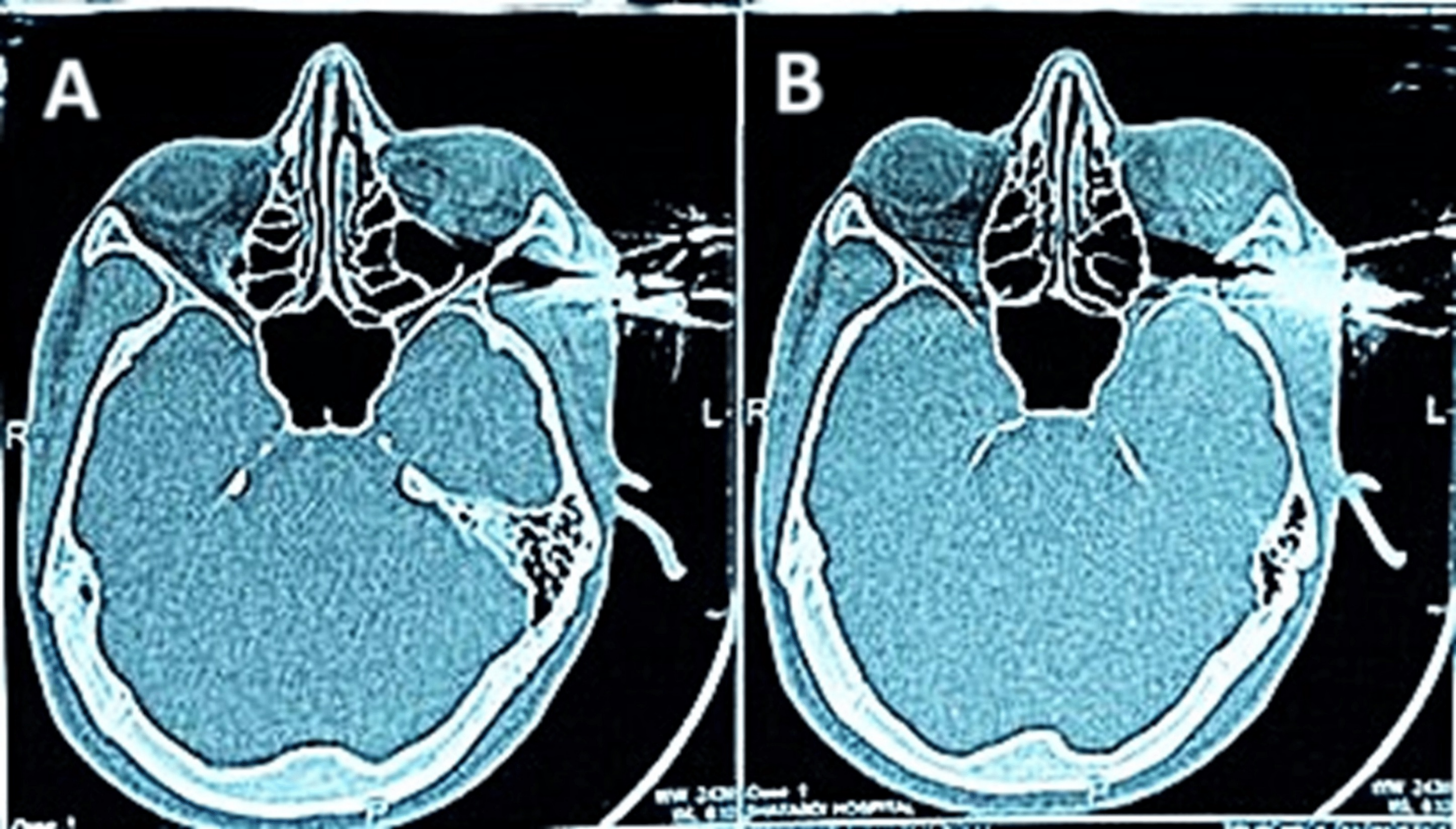
Computed tomography of Brain. (A) Metallic foreign body; (B) Metallic artifacts of foreign body and no intracranial penetration.

CT of the brain showed a metallic foreign body traversing through the posterior part of the left lateral orbital wall, posterior to the zygomatic process, and with the left optic nerve 2 mm medial to the tip of the bike key. There was no evidence of intracranial hemorrhage and no significant extension into the cranial cavity (Figure 3).

**Figure 4:**
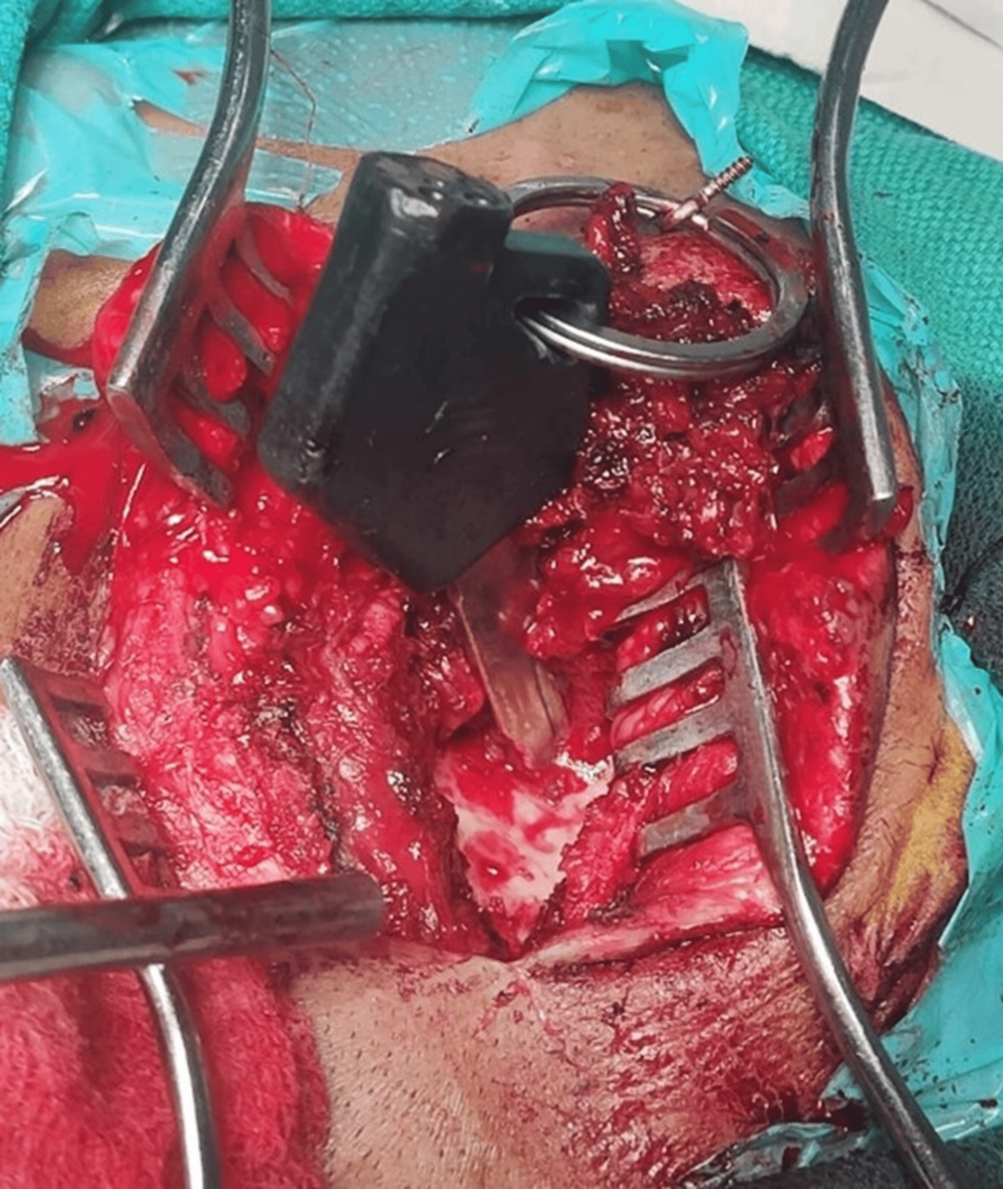
Intraoperative photographs showing bike key penetrating orbital wall.

**Figure 5:**
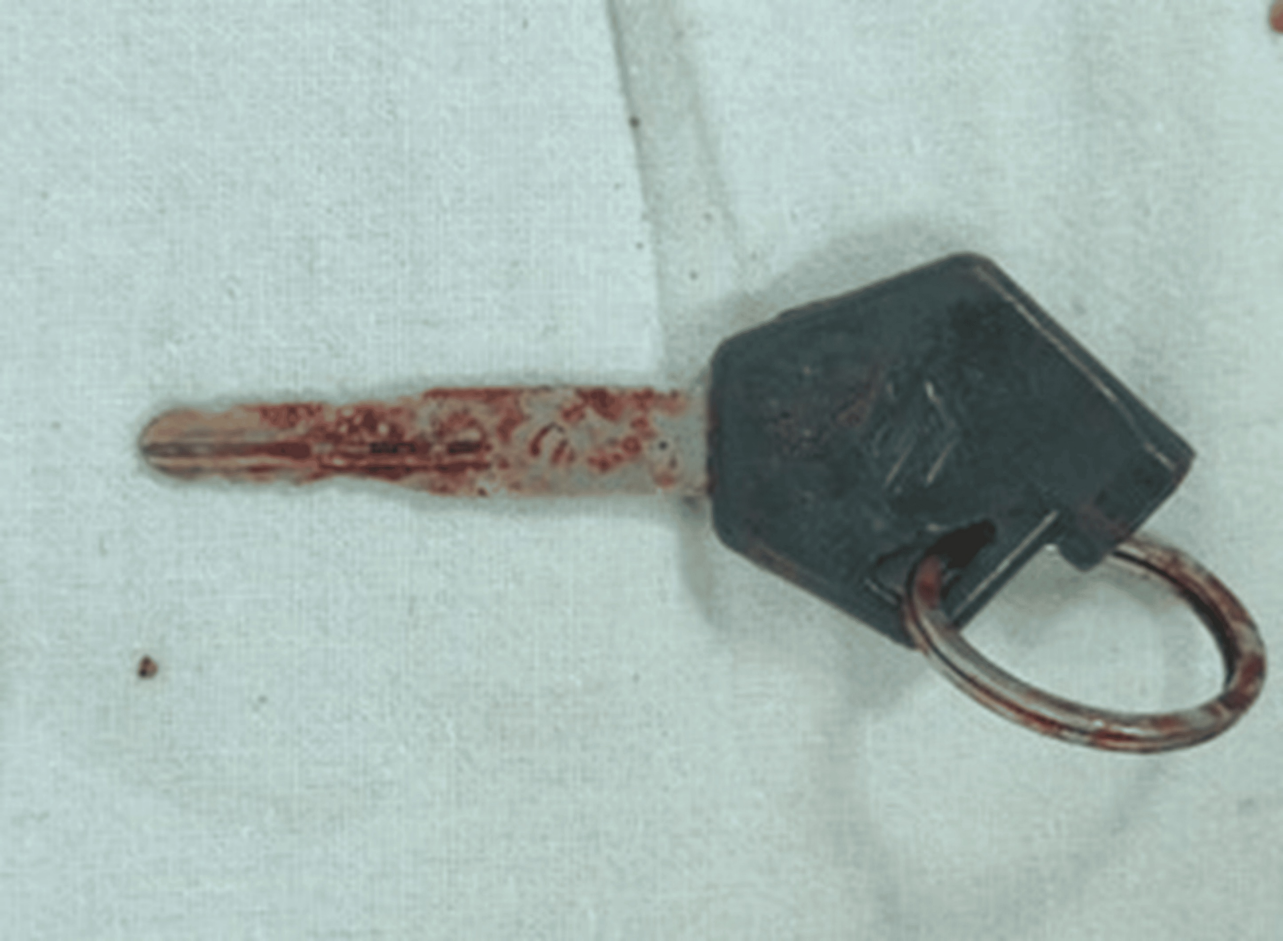
Removed bike key.

After pre-anesthesia assessment, the patient underwent left lateral wall orbitotomy with removal of a foreign body (bike key). The surgery was over within one hour, done by a neurosurgeon and general surgeon with an ophthalmologist on standby. A single burr hole over the temporal bone with an S-shaped incision was taken along both the upper and lower ends of the key. The distal end of the key was seen in the lateral orbital wall. Bone nibbling was done, and the key was removed from the lateral orbital wall. The key was not indenting into the brain parenchyma or eyeball and was safely removed without any complications (Figures 4, 5).

The patient was vitally stable after the procedure with no major post-operative neurological complications or ophthalmic complications. The patient was given intravenous antibiotics for a week. On post-operative day 10, sutures were removed. Postoperatively, the patient was advised to perform physiotherapy exercises in view of difficulty in opening the mouth and restriction of jaw movements, i.e., trismus. At one-month follow-up, the patient was well and had no complaints.

## Discussion

Incidence of penetrating head injuries is low, but they have a high risk of mortality and morbidity due to their association with injuries to brain structures, blood vessels, cranial nerves, and sometimes the eye. The consequences of penetrating brain injuries depend on the shape, range, force, velocity of the penetrating object, and location of the impact.

In this case, a bike key caused a penetrating head injury, which is an unusual and rare occurrence of a non-violent, low-velocity penetrating trauma. The management plan for such cases does not vary; it is similar to that of governing any other form of head injury [5]. A multidisciplinary approach, including an emergency trauma team, neurosurgeon, ophthalmologist, radiologists, anesthesiologists, and even a physiotherapy team in post postoperative course, is needed in such cases [6].

The best imaging modality for this type of head trauma is the non-contrast computed tomography, i.e., CT brain plain scan. In case of suspected vascular brain injuries, CT angiography plays a significant role. In the case of metallic foreign body imaging, CT produces metallic artifacts that can interfere with the visualization of the area of interest.

The major consequences of low-velocity stab wounds to the brain are hemorrhage and infection. MRI can be dangerous in the case of retained magnetic objects due to possible movement of the object in response to the magnetic torque [7].

The goals of surgical intervention in patients with these injuries are to remove the penetrating item from the brain parenchyma, in case of parenchymal involvement, also to remove necrotic tissue, debris and other potential contaminants, evacuation of any hematomas occurring from the injury and secure hemostasis, and to ensure watertight closure of the dura to prevent CSF leakage [8].

In this case, a mere bike key was turned into a weapon, which could cause grievous harm, but fortunately, it did not lead to any significant damage. The severity of injury depends on the size of the object, the site of impact, and the force of impact. The otherwise strong skull bone is thin in certain locations, such as the orbit. In this case, the tip of the key was just 2 mm away from the optic nerve, and just pulling it out could have caused damage [9,10]. One important “Don’t” is the removal of the object in the emergency department before imaging and preparation.

## Conclusions

Penetrating head injuries caused by everyday objects are rare and often present unique diagnostic and management challenges. This brief case report highlights that in penetrating head injury, the impaled object must not be removed even if there are no signs of neurological deficit in the patient at the time of presentation. Necessary investigations, such as a CT scan of the brain to locate its position, must be performed for the removal of the impaled object. All interventions for removal should be done by a multidisciplinary approach, in a controlled environment with adequate anesthesia. Reporting such unusual cases contributes valuable insights to the limited body of literature on atypical penetrating head injuries.
